# The Smad Dependent TGF-β and BMP Signaling Pathway in Bone Remodeling and Therapies

**DOI:** 10.3389/fmolb.2021.593310

**Published:** 2021-05-05

**Authors:** Ming-Li Zou, Zhong-Hua Chen, Ying-Ying Teng, Si-Yu Liu, Yuan Jia, Kai-Wen Zhang, Zi-Li Sun, Jun-Jie Wu, Zheng-Dong Yuan, Yi Feng, Xia Li, Rui-Sheng Xu, Feng-Lai Yuan

**Affiliations:** ^1^ Wuxi Clinical Medicine School of Integrated Chinese and Western Medicine, Nanjing University of Chinese Medicine, Wuxi, China; ^2^ Institute of Integrated Chinese and Western Medicine, The Hospital Affiliated to Jiangnan University, Wuxi, China; ^3^ Institute of Integrated Chinese and Western Medicine, The Third Hospital Affiliated to Nantong University, Wuxi, China

**Keywords:** transforming growth factor-β, bone morphogenetic proteins, bone remodeling, osteoporosis, Smad signaling pathway

## Abstract

Bone remodeling is a continuous process that maintains the homeostasis of the skeletal system, and it depends on the homeostasis between bone-forming osteoblasts and bone-absorbing osteoclasts. A large number of studies have confirmed that the Smad signaling pathway is essential for the regulation of osteoblastic and osteoclastic differentiation during skeletal development, bone formation and bone homeostasis, suggesting a close relationship between Smad signaling and bone remodeling. It is known that Smads proteins are pivotal intracellular effectors for the members of the transforming growth factor-β (TGF-β) and bone morphogenetic proteins (BMP), acting as transcription factors. Smad mediates the signal transduction in TGF-β and BMP signaling pathway that affects both osteoblast and osteoclast functions, and therefore plays a critical role in the regulation of bone remodeling. Increasing studies have demonstrated that a number of Smad signaling regulators have potential functions in bone remodeling. Therefore, targeting Smad dependent TGF-β and BMP signaling pathway might be a novel and promising therapeutic strategy against osteoporosis. This article aims to review recent advances in this field, summarizing the influence of Smad on osteoblast and osteoclast function, together with Smad signaling regulators in bone remodeling. This will facilitate the understanding of Smad signaling pathway in bone biology and shed new light on the modulation and potential treatment for osteoporosis.

## Introduction

Osteoporosis is a common systemic bone metabolism disorder characterized by decreased bone mass and disruption of the fine structure of bone tissue, which further results in increased bone fragility and occurrence of fracture (McGowan, 1993). Bone is a dynamic active tissue that needs to maintain the balance of bone mineralization and the integrity of bone structure through continuous remodeling (Lemaire et al., 2004). Continuous bone reconstruction is an important precondition of the preservation of bone health. Osteoclasts (OC) mediate the continuous absorption of bone matrix, followed by replacement of new bone by osteoblasts (OB). During bone remodeling, bone formation and bone resorption maintain a dynamic balance, which is called bone homeostasis (Kenkre and Bassett, 2018). Broken bone homeostasis would lead to osteoporosis. The mutual adjustment between OB and OC is the basis for maintaining bone homeostasis between bone formation and bone resorption. A variety of promising molecular signaling pathways are thought to be involved in this process, including MAPK, Wnt, Hedgehog, Notch, PI3K/Akt/mTOR, PDGF, IGF and Ca^2+^(Majidinia et al., 2018). It was previously shown that dysregulated Smad signaling pathway resulted in a number of bone disorders in humans (Liu et al., 2013).

The Smad protein family is an intracellular signaling protein identified in invertebrates by genetic screening methods in recent years (Ma and Meng, 2019). The name of Smad gene is a combination of the *Drosophila* gene ‘mothers against decapentaplegic' (Mad) and the *Caenorhabditis elegans* small protein (Sma) (Derynck et al., 1996). It has been shown that Smad protein is the key intermediates of canonical transforming growth factor-beta (TGF-β) signaling pathway and bone morphogenetic protein (BMP) pathway, which are important pathways that regulate bone homeostasis (Sánchez-Duffhues et al., 2015). It has been also suggested that Smad protein family and their activated downstream networks including the TGF-β pathway and the BMP pathway are concerned in cartilage development (Wang et al., 2020a). Therefore, understanding underlying Smad regulated molecular signaling pathways may profit the implications for osteoporosis. In this review, we attempted to shed light on recent studies of the effect of Smad signaling pathway in bone remodeling. Afterward we discuss the potential use of Smad for the treatment of osteoporosis.

## Introduction of Smad Protein

### Smad Protein

The Smad protein family is an intermediary molecule that transmits the signal generated by the binding of TGF-β and its receptor from the cytoplasm to the nucleus, thus playing an important role in signal transmission and regulating the transcription of downstream target genes (Luo, 2017).

Initially, through genetic screening of *drosophila*, it was discovered that the Mad polypeptide, which is downstream of the TGF-β family signaling pathway, has a highly conserved and unknown domain that was demonstrated to have high similarity with 3 types of peptides isolated from nematodes, cem-1, cem-2 and cem-3 (Sekelsky et al., 1995; Raftery and Sutherland, 1999). Since then, Mad has emerged as a new protein family. Then, through genetic screening in *Caenorhabditis elegans*, it was discovered that Sma-2, Sma-3 and Sma-4 all conserved domains that encode the same protein family at downstream of the TGF-β signaling pathway. The similarity of the structure and the connection with TGF-β family of Mad protein in *drosophila* and Sma protein in *elegans* attract people to homologous proteins in human, which was then named “Smad” in reference to its sequence similarity to the Sma and Mad proteins (Liu et al., 1996; Liu, 2003). And further studies also proved that Smad proteins act at downstream of serine/threonine kinase receptors (type I receptor and type II receptor), as well as Mad and Sma proteins (Urrutia et al., 2016).

### Members of Smad Family

**FIGURE 1:**
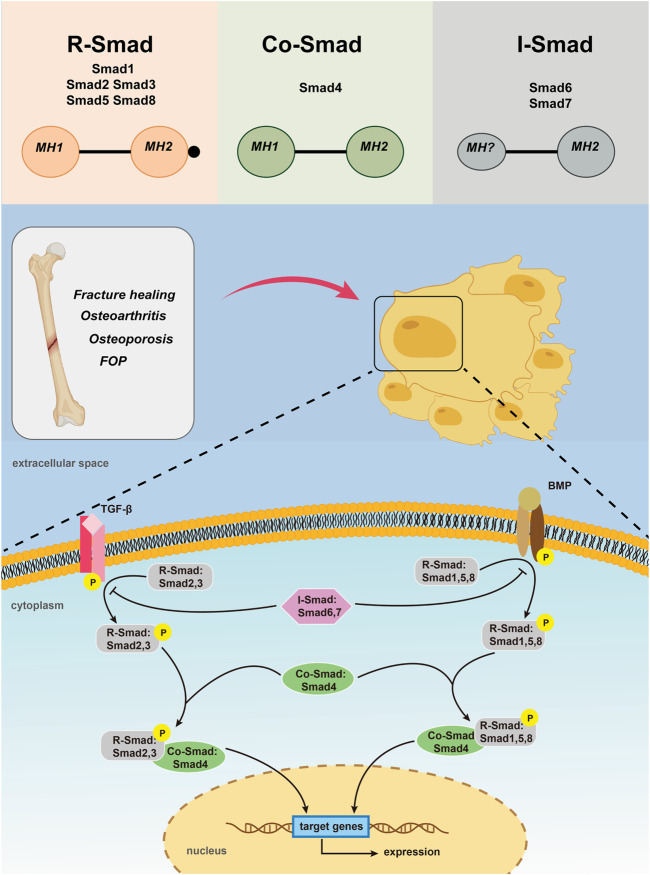
Activation of TGF-β/Smad and BMP/Smad pathways leading to Smad -mediated gene expression.

Up to now, 9 Smad members have been reported, which are called Smad 1- Smad 9 respectively. According to their structure and function, Smads can be divided into three categories. The first type are receptor-regulatory Smads (R-Smads) including Smad 1, 2, 3, 5, 8, 9, in which Smad 2 and Smad 3 are specific mediators of TGF-β or activin (Xu et al., 2003; Nakagawa et al., 2004; Li et al., 2011). They are phosphorylated between TGF-β and complexes formed between TGF-β type II receptors and activin receptor-like kinases ALK 4/5/7, or by activins and activin receptors 2, which participate in TGF-β and activin signaling pathways (Feng and Derynck, 2005; Katagiri and Tsukamoto, 2013). Complex formed by BMPs type I receptors ALK 1/2/3/6, BMPs type II receptors or activin receptors 2 could activate Smad 1, 5, 8 and possibly Smad 9, which is involved in conducting BMP signaling (Derynck and Zhang, 2003; Gomez-Puerto et al., 2019). Earlier studies have also confirmed that BMP-2/4 transmits signals via Smad 1, 5, and 8 (Zanotti et al., 2008; Song et al., 2011). The second type, the co-mediating Smad (Co-Smad), only one specie has been identified so far, namely the Smad 4. Smad 4 is like a central sensor, it can neither be phosphorylated nor bind to TGF-β receptors or BMP receptors. However, it can form heteromeric multimeric complexes with almost all activated R-Smads and therefore be able to participate in and regulate TGF-β signaling transduction (Wang et al., 2013). As Smad 4 has a proline-rich Smad 4 activiation domain (SAD) that regulates the interaction between transcriptional activation and inhibitory factors, which is necessary for transcriptional reactions that required for R-Smads activation (Kim and Jin, 2020). The third category is inhibitory Smads (I-Smads), with Smad 6 and Smad 7 included. In most resting cells, I-Smads are located in the nucleus. When stimulated by TGF-β or with overexpression of ubiquitination regulator Smurf 1, Smads 6 and 7 enter the cytoplasm from the nucleus (Chen et al., 2002). I-Smads bind to type I receptors, competitively interfere with recruitment and phosphorylation of R-Smads, and simultaneously inhibit the formation and activity of R-Smads and Co-Smad complexes (Kim and Jin, 2020). Smads 6, 7 can also interact with the ubiquitylated ligase Smurf E3, allowing it to bind to type I receptors, leading to receptor degradation to terminate signaling transduction (De Boeck and ten Dijke, 2012). I-Smads protein acts as a negative feedback signal for self-regulation of TGF-β signaling, as TGF-β can induce mRNA transcription of I-Smads. Therefore, I-Smads can tightly controll TGF-β signaling through negative feedback. What’s more, I-Smads can also inhibit Smad-mediated signal transduction, among which Smad 6 mainly inhibits BMP signal transduction, while Smad 7 inhibits TGF-β and BMP signal transduction (Figure 1) (Conidi et al., 2011; Zhang et al., 2014).

### Structural Characteristics of Smad Protein

Smad proteins are made up of two conserved domains: the Mad homolog domain 1 (MH1) in the N terminus that is essential for specific DNA binding and the Mad homolog domain 2 (MH2) in the C terminus that is responsible for protein-protein interactions. And the two domains are connected by a proline-rich nonconserved intermediate linking region(L) (Liu et al., 1996; Liu, 2003). MH2 is a functional effector region and is highly conserved in all Smad proteins, the functions of which include the interaction with type I receptors, form Smad polyplexes, and bind with other transcriptional coactivators or co-inhibitors. MH1 is a functional inhibitory region of MH2, which inhibits the function of MH2 when inactivated, and responsible for binding to a specific DNA sequence when activated. Unlike MH2, MH1 is only conserved in R-Smad and Co-Smad. MH1 and MH2 connected by L region together made up the smad protein (Guzman et al., 2012). When the receptor binds with the ligand, the two molecules dissociate to form a Smad complex that migrates to the nucleus, the nuclear accumulation of which causes transcription of the target gene. At present, the function of L region remains unclear.

### Smad Pathway

Although Smad pathway is present in most of the cell types and tissues, it is an additional pathway activated by TGF-β/BMP. In general, signal transduction of TGF-β and BMP ligands share many common principles. In the TGF-β pathway, when the TGF-β family ligand binds to the type II receptor, it activates the type II receptor kinase, and then phosphorylates the GS region. Before this occurs, type I receptor is catalytically inactive because the insertion of GS region into the kinase domain, dislocating the catalytic center. The phosphorylation of GS region directly leading to the phosphorylation of type I receptor, followed with R-Smads (Wrana et al., 1994; Huse et al., 2001). Phosphorylated R-Smads separate from type I receptors and form complexes with Smad 4 (Tao and Sampath, 2010). In the BMP pathway, BMPs eventually form a complex starting with the BMP type II receptor (BMPRII) kinase phosphorylating and then activating the type I receptor (BMPRI) kinase (Chan et al., 2007; Broege et al., 2013). The activated receptor complex further activates a series of downstream receptors that regulate R-Smads. During them, Smad 2 and Smad 3 are activated the TGF-β and activin receptors, whereas Smad 1, Smad 5 and Smad 8 are activated by ALK-1, ALK-2, ALK-3 and ALK-6. The phosphorylated R-Smads separated from the receptor and bind to Smad 4 to form the next complexes (Miyazono, 1999; Derynck and Zhang, 2003; Gomez-Puerto et al., 2019). These complexes formed through the TGF-β pathway and the BMP pathway are transferred to the nucleus and bind to different Smad-binding elements (SBEs), DNA transcription factors, transcriptional coactivators or co-inhibitors, and migrate into the nucleus (Conidi et al., 2013; Kopf et al., 2014). Their nuclear accumulation causes positive or negative regulation of target gene expression. For example, transcriptional co-activator with PDZ-binding motif (TAZ) is necessary for maintaining self-renewal markers in human embryonic stem cells and it is found that it is a key factor that controls the nuclear accumulation of heteromeric Smad 2/3–4 complexes. The loss of TAZ would directly lead to the failure of the complexes accumulating in the nucleus and thus inhibiting TGF-β signaling together with differentiating into a neuroectoderm lineage (Varelas et al., 2008). The N region of I-Smads is related to the specificity of other pathways and lacks MH1, which can compete with R-Smad for the type I receptor of TGF-β and BMPs, thereby surpressing the phosphorylation of R-Smad and inhibiting the Smad pathway (Bai and Cao, 2002).

## Role of Smad Family in Osteoporosis Through Transforming Growth Factor-β/Bone Morphogenetic Proteins Pathway

With the trend of global population aging, the incidence of osteoporosis has also increased year by year worldwide, which seriously threatens the health of elderlies. Osteoporosis is a disorder in which loss of bone strength leads to fragility fractures. Skeletal fragility can result from many reasons, while the only pathogenesis that can be relieved by treatment are excessive bone resorption and failure to failure to replace lost bone due to defects in bone formation (Raisz, 2005). Since OC is more active than OB, the tendency of bone resorption is always greater than that of bone formation. Therefore, regulating the balance between OB-mediated bone formation and OC-mediated bone resorption is the focus of treatment of osteoporosis (Appelman-Dijkstra and Papapoulos, 2015). Smad is directly involved in the induction of OB and OC formation and differentiation via the TGF-β/BMP pathway, which plays an integral role in the regulation of bone metabolism (Zhang et al., 2017).

### Smad Protein and Osteoblast

The OB responsible for bone formation activity is derived from bone marrow derived mesenchymal stem cells (BMSCs) (Endo and Mastumoto, 2014). OB proliferation and differentiation are regulated by a variety of growth factors, including TGF-β, BMP, and Smad. Both TGF-β and BMP pathways promote bone formation, and their signal transduction is both directly mediated by Smad (Chen et al., 2012).

#### Smad Protein and Osteoblast in Bone Morphogenetic Proteins Pathway

BMPs were first found as proteins that induce ectopic bone formation. After discovering the ectopic induction of decalcified bone, Urist et al. believed that it may contain an inducible factor that can induce the mesenchymal cells swimming around blood vessels to transform into irreversible bone line cells which can be used in bones. The factor was then proved and named "bone morphogenetic protein" (Urist, 1965; Urist and Strates, 1971). Since then, more and more researches on BMPs made people have a wider understanding of them. As multifunctional cytokines, they are now known to play important roles in an array of processes during formation and maintenance of various organs. Liu et al. found that BMPs are effective osteophytes that induce OB differentiation and bone formation and have been shown to induce bone formation in animals (Liu et al., 2013). BMP-2 induces the differentiation of BMSCs into OB and promotes bone formation by increasing the activation of alkaline phosphatase (ALP) and the expression of genes such as osteocalcin. Among all Smad proteins, Smad 1, Smad 5, and Smad 8 are closely associated with OB differentiation (J. H. Yang et al., 2016). After binding with BMP-I type A or type I B receptors, Smad 1, Smad 5, and Smad 8 are directly activated and phosphorylated, which then form heterotrimers or heterologous dimer with one or two R-Smads and one Smad 4 to enter the nucleus and acts on the gene sequence of OB-specific transcription factors such as runx 2 and osterix to up-regulate their expression (Tao and Sampath, 2010; Yang et al., 2013; Kim et al., 2014; Kopf et al., 2014). In addition, Smad 1 or Smad 5 specifically binds to the promoter of the PEBP 2αA/AML 3/CBFA 1 gene, the lack of which can leads to incapable of both endochondral and intramembranous bone formation (Komori et al., 1997; Takazawa et al., 2000). Some researchers have found that miR-155 is down-regulated after BMP-2 stimulation, which is capable of inhibiting differentiation of OB into osteocytes. The mechanism is to inhibit gene expression by binding to the 3′-UTR end of Smad 5 mRNA (Tsuji et al., 1998). Liu et al. believed that the ubiquitination of Smad 1/5 is responsible for the age related bone formation reduction, which lead them to Pleckstrin homology domain-containing family O member 1 (PLEKHO1), a molecule that could promote the ubiquitination of Smad 1/5. After the experiment on 50 bone samples of elderly patients with fractures, they found age-related increases in PLEKHO1 mRNA levels reduce phosphorylation of Smad 1/5 (p-Smad 1/5), and inhibit OB production. In addition, osteoblast-specific Smad 1 overexpression is beneficial to bone formation during aging, and which can be counteracted after overexpressing Plekho1 within osteoblasts(Liu et al., 2017).. Recent studies have found that the ligand DLL1 of the Notch signaling pathway promotes BMP9-induced osteogenic differentiation of BMSCs both *in vitro* and *in vivo*, which may be achieved by affecting multiple aspects of the BMP9 signaling pathway. DLL1 can promote the expression of BMP type I receptor ALK2, as well as up-regulate Smad 1/5/8 phosphorylation level and SBE transcriptional activity. Moreover, a recent study suggest that bone-forming peptide (BFP)-3, derived from the immature precursor of BMP-7 can cause osteogenic differentiation of bone marrow stromal cells by regulating the Smad 1/5/8 signaling pathways (Lee et al., 2018). These findings all indicate that Smad protein is involved in the BMP signaling pathway that induce OB formation in different degrees, and the formation of OB is closely associated with phosphorylated Smad.

#### Smad Proteins and Osteoblasts in the Transforming Growth Factor-β Pathway

In addition to the BMP pathway, Smad also promotes OB formation through the TGF-β pathway. Transforming growth factors-β is mainly stored as a latent complex in the extracellular matrix and exists in at least three isoforms: TGF-β1, TGF-β2, and TGF-β3. TGF-β1 deficient mice display reduced bone growth and mineralization (Geiser et al., 2005). TGF-β2 and TGF-β3 double knockout mice display a lack of distal parts of the rib (Dünker and Krieglstein, 2002). During signal transduction of this pathway, TGF-β firstly binds to its type II receptor and then activates its type I receptor. Activated type I receptors result in the phosphorylation of Smad 2 or Smad 3 at a C-terminal, which then forms complexes with Smad 4 and translocated into nucleus to bind to SBE on DNA through the hairpin-like structure of the MH1 region. The R-Smad-Smad 4 complex cooperates with sequence-specific transcription factors such as ymphoid enhancer-binding factor 1/T cell factor (LEF1/TCF), ymphoid enhancer-binding factor 1/T cell factor (LEF1/TCF), core-binding factor A/acute myeloneous leukemia (CBFA/AML) and the coactivators CREB-binding protein (CBP) or p300 to activate transcription in response to TGF-β ligand (Derynck and Zhang, 2003), thus affecting osteoblast proliferation, differentiation, and type I collagen synthesis (Runyan et al., 2012; Ota et al., 2013; Li et al., 2015). Researches suggest that OB and OC can communicate with each other through direct cell-cell contact, cytokines and extracellular matrix interaction (Chen et al., 2018). The connection between them and with outside is mediated by hemi-channels and gap junctions (GJ). And the communication between cells mediated by GJ is called gap junctional intercellular communication (GJIC), which plays an important role in the skeletal network, including participation in mechanical mechanotransduction, intracortical bone resorption and bone remodeling, regulating bone cell survival, etc (Plotkin et al., 2015; Xu et al., 2015). While the main component of GJ is connexin, during which connexin 43 (Cx43) is most highly expressed in bone, meaning it is closely related to the communication between OB and OC and the whole skeletal network. For exmple, Liu et al. found that the expression of Cx43 is related to the Smad-dependent TGF-β signaling pathway (Liu et al., 2018). The complex formed by Smad 2 or Smad 3 combined with Smad 4 can upregulate the expression of the target gene Cx43, thereby participating in the activities of bone cells, which indirectly verified the effect of Smad protein on bone cells in the TGF-β pathway again. Some researchers reported that puerarin promotes bone formation by stimulating the expression of Smad 2/3 mRNA and stimulating the secretion and synthesis of TGF-β1 (Okada et al., 2013). In addition, TGF-β can also induce self-expression of OB and induce BMP-2 expression in OB, enhancing its osteogenic capacity.

### Smad Protein and Osteoclasts

OCs responsible for bone resorption activity originate from hematopoietic stem cells. The formation of OC depends on the synergy of nuclear factor κB receptor activating factor ligand (RANKL) and colony stimulating factor 1 (CSF-1), both of which are secreted by OBs(Boyce, 2013). CSF-1 binds to its receptor activation signal pathway on the surface of immature OC and is essential for the proliferation and survival of OC precursors. RANKL promotes the formation, activation and survival of mature OC by binding to RANK on the surface of osteoclast precursor cells through both membrane-bound and soluble forms. They are essential for osteoclast formation and function because the mice lacking of them are found fail to form osteoclasts under homeostatic conditions, contributing to severe diseases such as osteopetrosis accompanied by a defect in tooth eruption (Dougall et al., 1999).

#### Smad Protein and Osteoclasts in Transforming Growth Factor-β Pathway

**FIGURE 2:**
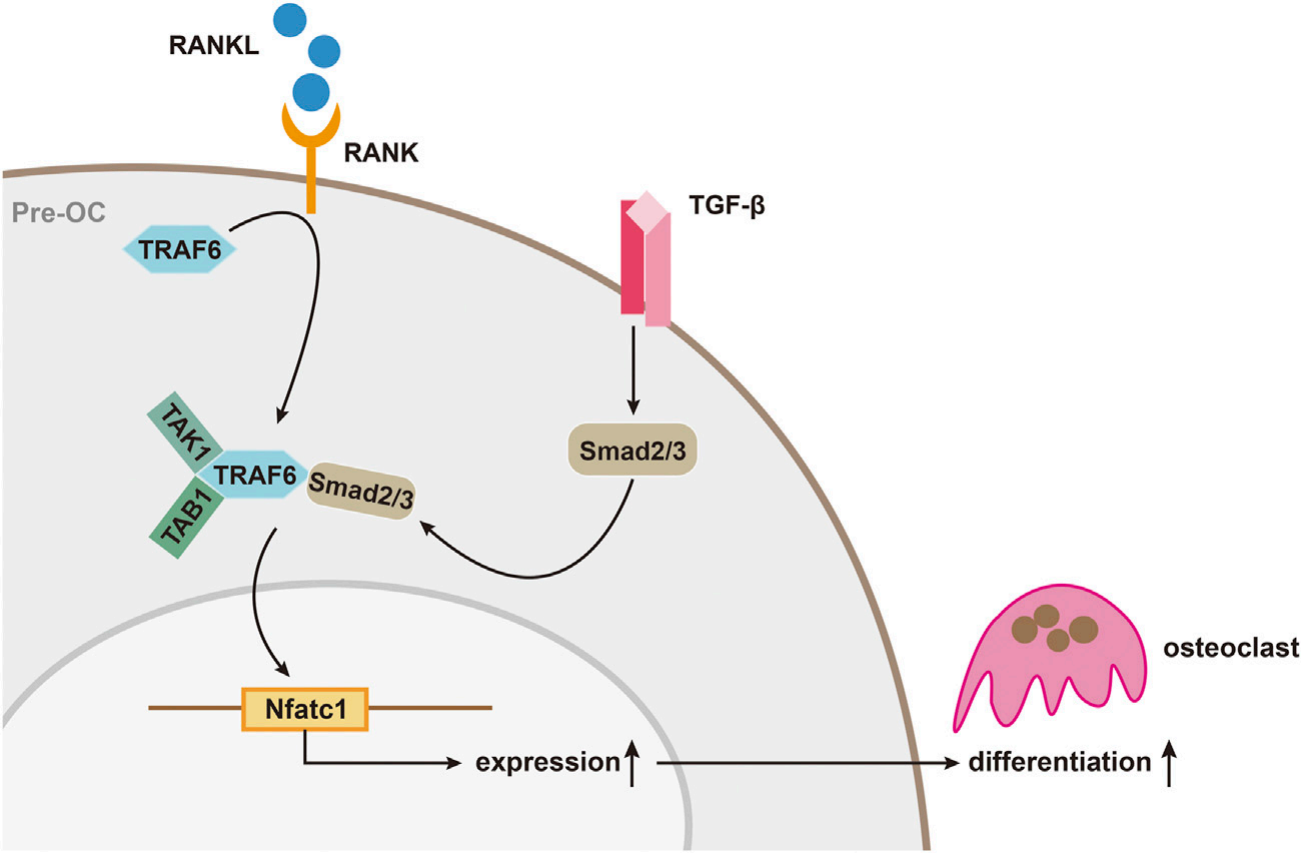
TGF-β/Smad signaling during osteoclast differentiation. The activation of Smad 2/3 by TGF-β, and Smad 2/3 binds directly to TRAF6-TAB1-TAK1 to form a complex that can promote RANKL-induced osteoclast differentiation.

Up to now, it is known that TGF-β is closely associated with the production and differentiation of OC. The differentiation of osteoclast is mainly mediated by RANKL/RANK pathway as RANKL would bind to macrophage colony stimulating factor to induce OC formation. While the existence of osteoprotegerin (OPG), a soluble decoy receptor can block osteoclast precursor differentiation by binding RANKL. The three of them maintain a certain balance until diseases such as osteoporosis occurs and inhibiting the RANKL/RANK pathway to inhibit osteoclast has been proven to be effective, which can also be reversely verified for the OPG knockout mice have osteoporosis (Mizuno et al., 1998; Nakashima and Takayanagi, 2011; Bae et al., 2017). In this process, on one hand, TGF-β was found to be able to directly act on bone marrow macrophages (BMMs) to promote the formation of OCs (Sriarj et al., 2015). On the other hand, it can also indirectly regulate the balance of RANKL with OPG to affect the differentiation of OC, which is complicated because TGF-β has not only been shown to stimulate OPG production in bone marrow stromal and osteoblastic cells (Takai et al., 1998), but also be thought to promote OC differentiation with the stimulation of RANKL (Yan et al., 2001; Fox and Lovibond, 2005; Sriarj et al., 2015). There are two main explanations for this contradictory effect in academia. One believes that it depends on the different model systems used *in vitro* experiments. The others balme on the different of the stage that TGF-β play its role as well as its concentration (Karst et al., 2004). In addition to that, the expression of downstream genes of the RANKL/RANK pathway is also inseparable from TGF and its activated Smad protein. Molecules such as TNF receptor-associated factor 6 (TRAF6), NF-κB, MAPK, and activator protein 1 (AP-1) have been identified as downstream mediators of RANK/RANKL signaling, and all of which are thought to influence osteoclasts through their effects on a common transcription factor, nuclear factor of activated T cell (NFATc1) (Nakashima and Takayanagi, 2011; Bae et al., 2017). NFATc1 has already been proved to be a key factor regulating OC differentiation because NFATc1-deficient embryonic stem cells cannot differentiate into osteoclasts and targeted disruption of Nfatc1 in hematopoietic cells in mice increases bone mass, with a marked decrease in osteoclasts (Takayanagi et al., 2002). While TGF-β can enhance RANKL-mediated translocation of complexes formed by Smad 2 or Smad 3 with Smad 4 into the nucleus and binds to the NFATc1 target gene, driving expression of NFATc1 (Fennen et al., 2016). In this process, binding of TRAF6 to the MH2 domain of Smad 3 is also important for the RANKL/RANK signal transduction (Fennen et al., 2016). Yasui et al. found that when the Smad pathway was blocked, induction of OC formation by RANKL was inhibited, but the activated mutant Smad 2 or Smad 3 could reverse this effect. Immunoprecipitation analysis revealed that Smad 2/3 directly forms a complex with TRAF6-TAB1-TAK1, which can stimulate RANKL and promote OC differentiation. Further analysis revealed that the MH2 region of Smad 3 was essential for the TRAF6-TAB1-TAK1 complex formation. TGF-β stimulates the activation of Smad 2 or Smad 3, and Smad 2 or Smad 3 binds directly to TRAF6-TAB1-TAK1 to form a complex that can promote RANKL-induced OC formation (Figure 2) (Yasui et al., 2011).

#### Smad Proteins and Osteoclasts in the Bone Morphogenetic Proteins Pathway

Among BMPs, BMP-2 is closely associated with the formation of OC, also stimulates the bone resorption activity of OC. BMP-2 mainly induces the expression of CSF-1 to promote OC formation (Sun et al., 2014). It also mediates Smad of the BMP pathway to synergize with the CREB-binding protein to increase the expression of CSF-1, and the up-regulation of CSF-1 further promotes OC production (Mandal et al., 2009). In RANKL/RANK pathway, RANKL and BMP-2 can increase their expression under the action of interleukin-1α to promote OC formation, improve the survival rate of OC, and promote the differentiation of OC(Liu et al., 2005). Nevertheless, BMP-2 alone cannot improve the survival rate of OC. Yoshikawa et al. (Yoshikawa et al., 2015)found that BMP-2-induced RANKL mRNA expression was inhibited in the primary Smad 1 knockout OB, resulting in decreased OC formation. In addition to Smad 2 and Smad 3 responsible for mediating TGF-β signaling pathways and Smad 1 and Smad 5 responsible for mediating BMP pathways, Smad 4, as a Co-Smad binds to R-Smads that are phosphorylated by TGF-β/BMP stimulation, also paticipate in the formation of OC as a matter of course. Amy et al. found that after knockout of Smad 1, 4 and 5, respectively, bone resorption activity of OC decreased, and the expression of OC differentiation markers was also decreased, indicating that expression of Smad 1, 4 and 5 is necessary for the differentiation of OC(Tasca et al., 2015). In addition, Li et al.used mice with exon 1 of the Smad 7 gene knocked out to mimic the functional loss of Smad 7. Compared with normal mice, there was no significant difference in the morphology and length of the metaphysis of the left femur, but bone mass and trabecular bone were lower than those of the control group. More OCs were found under the microscope when compared with the control group. This shows that partial loss of Smad 7 function inhibits bone formation and promotes bone resorption, suggesting the promotion of Smad 7 to osteoclast formation (Li et al., 2014).

## Therapeutic Potential of Smad Signaling Regulators for Treatment of Osteoporosis

Smad has major effects on the induction of OB and OC production and differentiation through TGF-β/BMP pathway under various circumstances, which plays a significant role in the regulation of bone metabolism. Although precise regulatory mechanisms are not well understood, inhibitors and activators targeting the Smad signaling pathway can certainly produce specific effects on the treatment of osteoporosis. Table 1 lists a range of signaling pathway inhibitors for popular targets. For example, since the Smad pathway activated by TGF-β pathway is initiated by phosphorylation of type I receptors, small molecule inhibitors targeting TGFI type kinase domain can inhibit the activation of the Smad pathway (Wang et al., 2020c). Scios (Johnson & Johnson) developed a small molecule, orally active TGFI type kinase inhibitor called SD-208, which can block the TGF-β-induced phosphorylation of the Smad 2/Smad 3 in a dose-dependent manner (Wang et al., 2020b). Experiments by Mohammad et al. in mice proved this point and the results showed that SD-208, an inhibitor that blocks the TGF-Smad pathway can prevent Experiments by Mohammad et al. in mice proved this and the results showed that SD-208, an inhibitor that blocks the TGF**-**β-Smad signaling pathway can prevent the development and progression of melanoma bone metastases (Mohammad et al., 2011). Wei et al. used the SBE-bla assay as a novel first-tier screen to determine whether a chemical has the potential to inhibit TGFβ1-induced Smad 2/3/4 signaling, which provides us train of thought that more chemicals can therefore be discovered and put into test in bone remodeling (Wei et al., 2019). In addition, there are some known TGF-β/Smad inhibitors having been put into clinical practice such as pirfenidone and haloperidol hydrochloride, which having shown to be effective. While in BMP-Smad pathway, blocking Smad 1/5/8 phosphorylation with dorsomorphin is proved to prevent terminal differentiation and mineralization of cartilage tissue while sustaining further cartilagematrix production (Hellingman et al., 2011). Similarly, it reminds us that compounds or drugs which is able to activate Smad signaling pathway through TGF-β or BMP pathway have a adjusting function to osteoporosis (Wang et al., 2020d). Of course, the direction ultimately depends on the specific cells they regulate. Therefore, we also summarize a list of activators of Smad pathway (Table 2), hoping that experiments on function of these compounds in osteoporosis can be carried out. However, since the TGF/BMP pathway is ubiquitous in the human body, the regulation of one place is likely to involve another. For example, when considering promoting osteoblast formation through the TGF-β pathway, TGF-β activation in bone tendon insertion would induce enthesopathy-like disease should also be considered (Wang et al., 2018). Related regulatory mechanisms should be studied more precisely, and the location and concentration of the drug should also be limited to avoid side effects.

**TABLE 1:** Inhibitors of Smad signaling pathway.

Compounds	Pathway	Targets	References(s)
A-77–01	TGF-β - Smad pathway	TGF-βRI	Wang et al. (2020a)
A-83–01	TGF-β - Smad pathway	TGF-βRI	Wang et al. (2020a)
BT173	TGF-β - Smad pathway	TGF-βRI	Liu et al. (2017)
Dorsomorphin	BMP-SMAD pathway	BMPRI	Sakata and Chen (2011)
EW-7197	TGF-β - Smad pathway	TGF-βRI	Wang et al. (2020a)
FKBP12	BMP-SMAD pathway	ALK2	Sakata and Chen (2011)
	TGF-β - Smad pathway	TGF-βRI	
GW788388	TGF-β - Smad pathway	TGF-βRI	Wang et al. (2020a)
Heparins	BMP-SMAD pathway	Unknow	Silvestri et al., (2019)
IN-1130	TGF-β - Smad pathway	TGF-βRI	Wang et al. (2020a)
Isorhamnetin	TGF-β - Smad pathway	Unknow	Yang et al. (2016b)
K02288	BMP-SMAD pathway	ALK2	Ali and Brazil (2014)
LDN193189	BMP-SMAD pathway	BMPRI	Silvestri et al. (2019)
LY2109761	TGF-β - Smad pathway	TGF-βRI	Wang et al. (2020a)
LY2157299	TGF-β - Smad pathway	TGF-βRI	Wang et al., (2020a)
LY3200882	TGF-β - Smad pathway	TGF-βRI	Wang et al. (2020a)
LY364947	TGF-β - Smad pathway	TGF-βRI	Wang et al. (2020a)
Momelotinib	BMP-SMAD pathway	ALK2	Silvestri et al. (2019)
PFD/FD	TGF-β - Smad pathway	unknow	Wang et al. (2020a)
Progesterone	TGF-β - Smad pathway	TGF-βRI	Kunzmann et al. (2018)
R-268712	TGF-β - Smad pathway	TGF-βRI	Wang et al. (2020a)
RepSox	TGF-β - Smad pathway	TGF-βRI	Wang et al. (2020a)
SB431542	TGF-β - Smad pathway	TGF-βRI	Wang et al. (2020a)
SB-505124	TGF-β - Smad pathway	TGF-βRI	Wang et al. (2020a)
SB-525334	TGF-β - Smad pathway	TGF-βRI	Wang et al. (2020a)
SD-208	TGF-β - Smad pathway	TGF-βRI	Wang et al. (2020a)
SIS3	TGF-β - Smad pathway	TGF-βRI	Li et al. (2010)
Testosterone	TGF-β - Smad pathway	TGF-βRI	Sakata and Chen (2011)
TMPRSS6	BMP-SMAD pathway	ALK2, ALK3	Silvestri et al. (2019)
TP-0427736	TGF-β - Smad pathway	TGF-βRI	Wang et al. (2020a)
Valproic acid	TGF-β - Smad pathway	TGF-βRI	Qi et al. (2019)

FKBP12: FK506-binding protein 12; PFD: Pirfenidone; FD: fluorofenidone; TMPRSS6: transmembrane serine protease 6; TGF-βRI: TGF-β receptors type I; BMPRI: BMP type I receptor.

**TABLE 2:** Activators of smad signaling pathway.

Compounds	Pathway	Targets	References(s)
Activin-A	TGF-β - Smad	TGF-βRI	Kajita et al. (2018)
Bacitracin	BMP-SMAD pathway	BMP2	Li et al. (2018)
CP	BMP-SMAD pathway	BMP2	Jadai et al. (2019)
Daidzein	TGF-β - Smad	TGF-βRI	Zhao et al. (2015); Hu et al. (2016)
	BMP-SMAD pathway		
Dexmedetomidine	BMP-SMAD pathway	Unknow	Shan et al. (2018)
Myricetin	BMP-SMAD pathway	BMP2	Kim et al. (2018)
Nano Nio	TGF-β - Smad	TGF-βRI	Zhang et al. (2020)
Rapamycin	TGF-β - Smad	TGF-βRI	(Osman et al. (2009); Lee et al. (2010)
	BMP-SMAD pathway	ALK2	
SALL4	TGF-β - Smad	TGF-βRI	Zhang et al. (2018)
Tacrolimus	BMP-SMAD pathway	ALK2	Chaikuad et al. (2012)
Vaspin	Smad-Runx2	Smad 2/3	Wang et al. (2020b)
Vitamin D	BMP-SMAD pathway	BMP2	Jadai et al. (2019)

CP: Cowpea isoflavones; Nano NiO: nickel oxide nanoparticles.

## Prospect of Smad in Osteoporosis Research

In conclusion, pathogenesis of osteoporosis is mainly an imbalance of bone homeostasis between OB-mediated bone formation and OC-mediated bone resorption (Yang and Duan, 2016). Smad directly participates in the induction of OB and OC production and differentiation through TGF-β/BMP pathway under different circumstances, which plays a significant role in regulating bone metabolism (Liu et al., 2014). However, the current research on Smad is still at early stage. The researches on bone formation and bone resorption are mainly limited to animal studies, with few direct study on osteoporosis patients. The precise regulation mechanism of Smad is also not fully understood. Smads is involved in regulation of TGF-β/BMP pathway, and contribute to the formation of both OB and OC. It has not been fully understood that which factors can induce Smad to promote OB formation, and which factors can induce Smad to promote OC formation. Further relevant studies are necessary to explore the specific mechanisms and influencing factors of Smad’s role in bone formation and bone resorption. How to promote the formation of OB while avoiding the formation of OC will be a hot topic for future research on drugs for the prevention and treatment of osteoporosis.
